# Therapeutic potential of mesenchymal stem cells from human iPSC‐derived teratomas for osteochondral defect regeneration

**DOI:** 10.1002/btm2.10629

**Published:** 2023-11-29

**Authors:** Jiseong Kim, Jin‐Su Kim, Dohyun Kim, Alvin Bacero Bello, Byoung Ju Kim, Byung‐Hyun Cha, Soo‐Hong Lee

**Affiliations:** ^1^ Department of Biomedical Technology Dongguk University Goyang‐si Republic of Korea; ^2^ Department of Biomedical Science CHA University Seongnam‐si Republic of Korea; ^3^ Biomaterials Research Center CELLINBIO Co., Ltd. Suwon‐si Gyeonggi‐do Republic of Korea; ^4^ Department of Integrative Engineering Chung‐Ang University Seoul Republic of Korea; ^5^ Department of Rearch & Development team ATEMs Seoul Republic of Korea; ^6^ Division of Biomedical Convergence College of Biomedical Science, Kangwon National University Chuncheon‐si Republic of Korea

**Keywords:** condensed MSC mass, human induced pluripotent stem cells, mesenchymal stem cells, teratomas

## Abstract

Human induced pluripotent stem cells (iPSCs) hold great promise for personalized medicine, as they can be differentiated into specific cell types, especially mesenchymal stem cells (MSCs). Therefore, our study sought to assess the feasibility of deriving MSCs from teratomas generated from human iPSCs. Teratomas serve as a model to mimic multilineage human development, thus enriching specific somatic progenitors and stem cells. Here, we discovered a small, condensed mass of MSCs within iPSC‐generated teratomas. Afterward, we successfully isolated MSCs from this condensed mass, which was a byproduct of teratoma development. To evaluate the characteristics and cell behaviors of iPSC‐derived MSCs (iPSC‐MSCs), we conducted comprehensive assessments using qPCR, immunophenotype analysis, and cell proliferation‐related assays. Remarkably, iPSC‐MSCs exhibited an immunophenotype resembling that of conventional MSCs, and they displayed robust proliferative capabilities, similar to those of higher pluripotent stem cell‐derived MSCs. Furthermore, iPSC‐MSCs demonstrated the ability to differentiate into multiple lineages in vitro. Finally, we evaluated the therapeutic potential of iPSC‐MSCs using an osteochondral defect model. Our findings demonstrated that teratomas are a promising source for the isolation of condensed MSCs. More importantly, our results suggest that iPSC‐MSCs derived from teratomas possess the capacity for tissue regeneration, highlighting their promise for future therapeutic applications.

**Translational Impact Statement**

To overcome the limitations associated with conventional methods for MSCs derivation and differentiation from PSCs, we suggested a novel method to utilize teratomas as a potential source of MSCs. We found condensed MSCs mass inside of the teratomas and isolated iPSCs‐MSCs from the condensed mass. Our method is closer to naïve MSC derivation in the development of human embryos. Therefore, we are certain that MSCs derived in a teratoma environment will be superior to those derived in a conventional environment of an in vitro culture.

## 1 INTRODUCTION

Human pluripotent stem cells (PSCs) encompass human embryonic stem cells (ESCs)^1^ and human induced pluripotent stem cells (iPSCs).^2^ These cells possess self‐renewal characteristics that provide an unlimited source of cells and the potential to differentiate into the three primary germ layers, thus giving rise to most cell types found in the human body.^3^ Therefore, many researchers have focused their efforts on developing therapeutics using organ‐specific cells derived from either ESCs or iPSCs.^4^ Recent research has indicated that there are no significant differences in RNA expression^5^ and patterns of histone modification^6^ between ESCs and iPSCs. Nevertheless, cells derived from iPSCs offer several advantages over those derived from ESCs. First, iPSC reprogramming does not require the use of a human embryo, eliminating ethical concerns associated with ESCs. Additionally, iPSCs can be a superior choice for disease modeling because they naturally harbor mutations similar to those found in patients.^7^ Similarly, cells derived from iPSCs are associated with fewer immune reactions^8^ than those from ESCs. Furthermore, iPSCs retain an epigenetic memory of their tissue of origin, which enables the customization of iPSCs to target each patient's specific disease, thereby advancing cell therapy prospects.^9^

Mesenchymal stem cells (MSCs), a type of adult stem cell, are widely acknowledged as a valuable resource for clinical cell therapy. Several advantages make them attractive for clinical use. For example, MSCs can be easily obtained from a variety of tissues, such as fat, bone marrow, and cord blood.^10^ Furthermore, MSCs possess the potential to differentiate into mesodermal tissue lineages, including osteocytes, chondrocytes, and adipocytes.^11^ However, with the aid of various cytokines and growth factors, they can also differentiate into numerous cell types, primarily those of ectodermal and endodermal lineages.^12^ This broad differentiation potential makes MSCs a valuable tool for researchers exploring tissue regeneration. MSCs are renowned for their neuroprotective,^13^ anti‐inflammatory,^14^ and immunomodulatory effects.^15^ Given these properties, MSCs have been investigated for their potential in tissue regeneration and stem cell therapy, employing both autologous and allogeneic stem cell transplantation. The initial therapeutic applications using MSCs were reported in the context of bone and cartilage regeneration.^16^, ^17^ Over the past few decades, MSCs have emerged as a crucial component in the regeneration of bone, both in vitro and in vivo. Furthermore, MSCs have been employed to treat a variety of conditions, including myocardial infarction,^18^ kidney injury,^19^ diabetes,^20^, ^21^, ^22^ Parkinson's disease,^23^ and stroke.^24^, ^25^ Despite the many advantages of tissue‐derived MSCs for clinical trials, their implementation poses several disadvantages for clinical trials such as limitations in human tissue sources, low yield, and population heterogeneity.^26^ To address these limitations, MSCs can be derived not only from various tissues in the body but also from PSCs, offering an unlimited cell source.^4^ Various methods for deriving and isolating MSCs from ESCs or iPSCs have been developed, involving co‐culture with OP9 cells,^27^ embryonic body formation,^28^ growth factors,^29^ chemical cues,^30^, ^31^ extracellular matrix,^32^ and histone methylation.^33^ Unfortunately, these methodologies typically involve long‐term culture (approximately 30 days), necessitating significant resource consumption in the form of growth factors, cytokines, biomaterials, and media. Furthermore, they are prone to xeno‐contamination when employing other animal feeder cells. Moreover, these methods still fall short of clinical applicability due to their low isolation efficiency, limited differentiation capability, heterogeneous cell populations, and high costs.^32^

To overcome the limitations associated with PSCs‐derived MSCs, our study sought to identify a novel source of MSCs that accurately mirrors the microenvironment found in various tissues throughout the human body. Recently, several studies have reported the use of teratomas generated from mouse PSCs as a source for isolating skeletal muscle stem cells,^34^ neural stem cells,^35^, ^36^ and hematopoietic stem cells.^37^ These studies have demonstrated that teratomas are valuable resource for emulating multi‐lineage human development and enriching specific somatic progenitor and stem cells.^38^ These distinctive attributes offer compelling evidence of the potential use of teratomas as a source of MSCs. Therefore, we hypothesized that teratomas generated from iPSCs have the capacity to yield a concentrated mass of mesodermal progenitor cells or MSCs suitable for therapeutic applications in tissue regeneration. Remarkably, current methods have never harnessed teratomas derived from human ESCs or iPSCs as in vivo bioactuators for the derivation or isolation of MSCs. Therefore, our study is the first to propose that iPSCs‐generated teratomas can serve as a viable source of MSCs for tissue engineering.

In this study, we demonstrate that MSCs can indeed be isolated from the small, condensed MSC mass within iPSCs‐generated teratomas. These iPSC‐derived MSCs exhibit cell characteristics similar to those of conventional adult MSCs, such as adipose‐derived stem cells (ASCs) and bone marrow‐derived MSCs (BM‐MSCs). Collectively, these findings strongly suggest that teratomas generated from iPSCs have the potential to yield condensed MSC masses, which can be isolated and hold promise as a cell source for bone regeneration therapies.

## 2 RESULTS

### 2.1 Characterization of condensed MSC mass isolated from the inside of human iPSC‐generated teratomas

**FIGURE 1:**
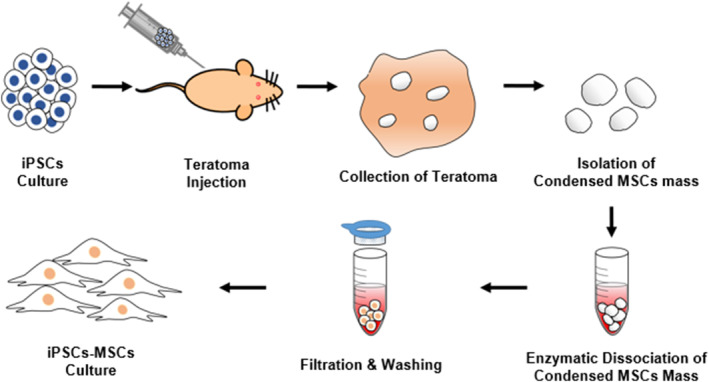
Schematic diagram depicting the entire isolation process of iPSCs‐derived MSCs from teratomas.

**FIGURE 2:**
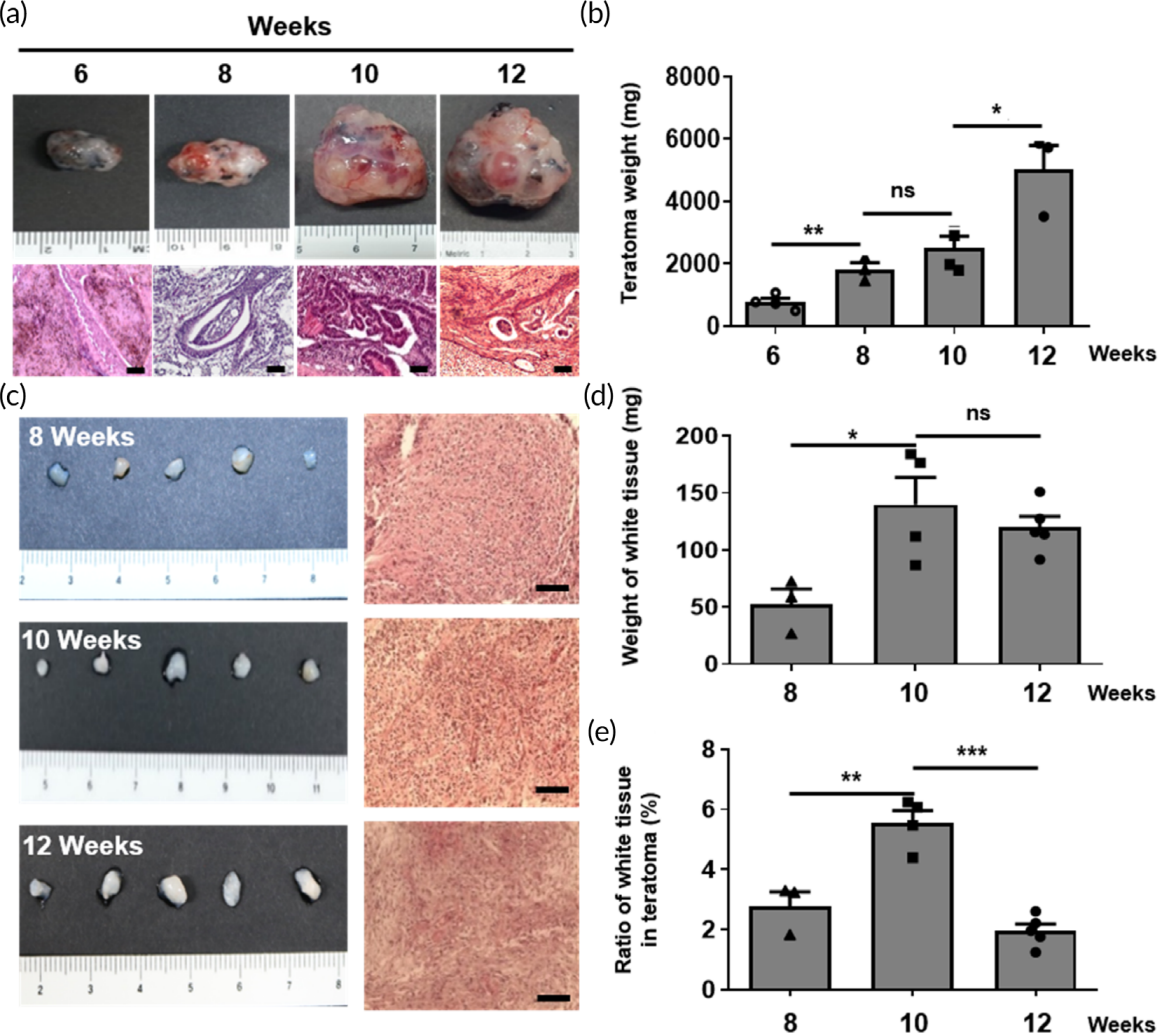
Characterization of teratomas and condensed MSC mass from human iPSC‐derived teratomas during teratoma development. (a) Macroscopic appearance and histological examination of teratomas as a function of post‐implantation time (weeks). Scale bar, 100 μm. (b) Comparison between the weights of teratomas as a function of post‐implantation time (weeks). The data are presented as the means ± s.d. (*n* = 6, biologically independent samples). *p < 0.05, **p < 0.01, ***p < 0.001, ns: no significance; based on one‐way ANOVA followed by Tukey's post hoc test. (c) Macroscopic appearance and histological examination of condensed MSC mass. Scale bar, 100 μm. (d) Comparison between the weights of condensed MSC mass. Data are presented as the means ± s.d. (*n* = 6, biologically independent samples). *p < 0.05, **p < 0.01, ***p < 0.001, ns: no significance; based on one‐way ANOVA followed by Tukey's post hoc test. (e) Comparison of the ratio of condensed MSC mass inside of the teratomas depending on the weeks post‐implantation. The data are presented as the means ± s.d. (*n* = 6, biologically independent samples). *p < 0.05, **p < 0.01, ***p < 0.001, ns: no significance; based on one‐way ANOVA followed by Tukey's post hoc test.

To explore the potential applicability of teratomas generated from human PSCs as a source of MSCs, we subcutaneously injected human iPSCs, a promising PSC source, into the dorsal flank of immunodeficient mice to induce the formation of teratomas (Figure 1). Afterward, we harvested these teratomas at 6, 8, 10, and 12 weeks and verified the presence of the three germ layers in them (Figure 2a). We then monitored the growth of the teratomas by assessing their weight (Figure 2b), observing an increase from 7 to 800 mg at 6 weeks to 5000 mg at 12 weeks. During this process, we identified several small, condensed, and semi‐solid tissues within the teratomas, which were absent at the 6‐week mark. Intriguingly, histological sections revealed that these tissues remained uniform from week to week and were comprised of a single cell type (Figure 2c). The quantity of these small, condensed tissues within the teratomas was measured based on their weight and showed a threefold increase at 10 weeks compared to 8 weeks. However, there was no statistically significant difference between 10 weeks and 12 weeks (Figure 2d). We also calculated the proportions of each type of small condensed tissue relative to the total weight of the teratomas and the small condensed tissues extracted from them. Based on these ratios, our findings confirmed that these small condensed tissues could be efficiently collected at 10 weeks (Figure 2e).

### 2.2 Cells isolated from the small condensed tissues exhibit MSC characteristics and show higher proliferative capacities

**FIGURE 3:**
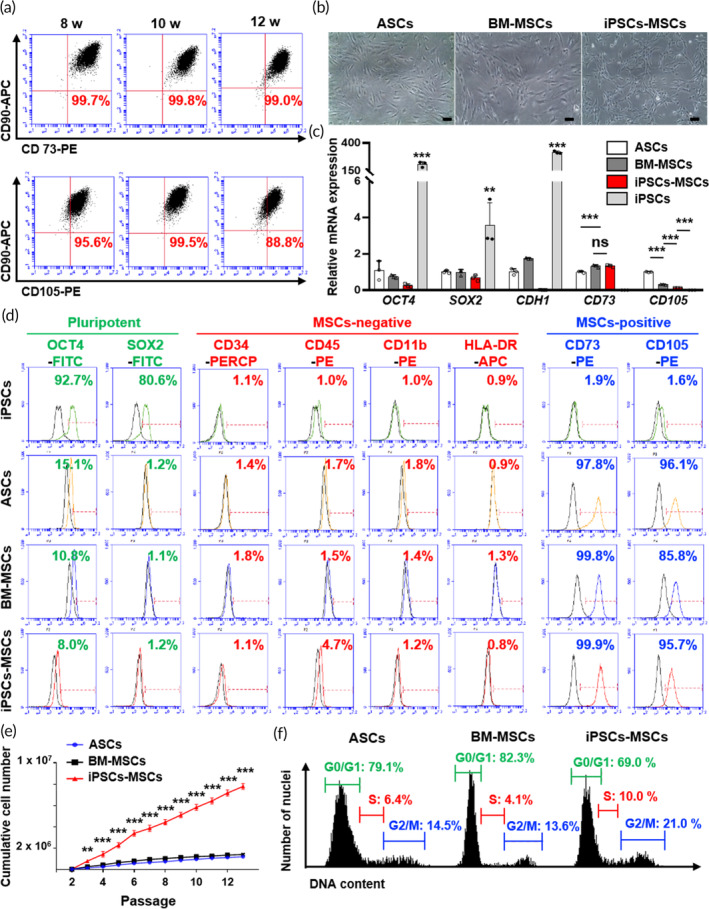
Characterization of condensed MSC mass‐derived mesenchymal stem cells (iPSCs‐MSCs). (a) Immunophenotype analysis of iPSCs‐MSCs isolated at 8, 10, and 12 weeks with MSCs markers CD73, CD90, and CD105 through flow cytometry. In every group, CD73 and CD90 double‐positive cells were higher than 99%. CD90 and CD105 double‐positive cells were approximately 90%. (b) Assessment of the morphology of ASCs, BM‐MSCs, and iPSCs‐MSCs via microscopy. Scale bar, 100 μm. (c) Comparison of mRNA expression of pluripotent markers (*OCT4, SOX2, and CDH1*) and MSC markers (*CD73*, *CD105*) between ASCs, BM‐MSCs, iPSCs‐MSCs, and iPSCs. The data are presented as the means ± s.d. (*n* = 3, biologically independent samples). *p < 0.05, **p < 0.01, ***p < 0.001, ns: no significance; based on one‐way ANOVA followed by Tukey's post hoc test. (d) Immunophenotype analysis of pluripotent markers (OCT4, SOX2), MSC‐negative markers (CD34, CD45, CD11b, and HLA‐DR), and MSC‐positive markers (CD73 and CD105) between iPSCs, ASCs, BM‐MSCs, and iPSCs‐MSCs through flow cytometry. (e) Measurement and comparison of the cumulative cell number until passage 13. The data are presented as the means ± s.d. (*n* = 3, biologically independent samples). *p < 0.05, **p < 0.01, ***p < 0.001, ns: no significance; based on one‐way ANOVA followed by Tukey's post hoc test. (f) Flow cytometry plot showing the cell cycle analysis of ASCs, BM‐MSCs, and iPSCs‐MSCs with DNA contents.

Flow cytometry analysis was conducted using cells isolated from the dissociated small, condensed tissues by collagenase. Remarkably, iPSCs‐derived MSCs (iPSCs‐MSCs) obtained from these small condensed tissues exhibited a higher expression of positive MSC markers, such as CD73, CD90, and CD105 (Figure 3a). Additionally, they displayed a cell morphology similar to that of ASCs and bone marrow‐derived mesenchymal stem cells (BM‐MSCs), as depicted in Figure 3b. To provide a comprehensive assessment of the potential of iPSCs‐MSCs in comparison to conventional MSCs (including iPSCs as the negative control group), we examined their endogenous molecular characteristics. Particularly, the mRNA levels of target genes were assessed to confirm pluripotent/MSC markers, revealing that iPSCs‐MSCs closely resembled ASCs and BM‐MSCs, showing significantly lower expression of pluripotent markers such as *OCT4*, *SOX2*, and *CDH1* (the gene encoding E‐cadherin) and higher expression of the MSC marker *CD73*, when compared to iPSCs (Figure 3c). Furthermore, iPSCs exhibited higher expression of pluripotent markers such as OCT4 and SOX2 but lower expression of MSC‐positive markers such as CD73 and CD105, as well as MSC‐negative markers like CD34, CD45, CD11b, and HLA‐DR (Figure 3d). We also quantified the cumulative cell numbers for ASCs, BM‐MSCs, and iPSCs‐MSCs (illustrated in Figure 3e). During the assay, the number of iPSCs‐MSCs exhibited a significant increase with each passage, maintaining their proliferative capacity. In contrast, the numbers of ASCs and BM‐MSCs gradually declined, eventually leading to senescence after 6 or 7 passages. Additionally, we employed PI staining to observe the cell cycle at passage 5 for each cell group (depicted in Figure 3f). This observation indicated that iPSCs‐MSCs had a more active cell cycle with higher G‐2 phase and S‐phase proportions compared to ASCs and BM‐MSCs. Furthermore, iPSCs‐MSCs maintained these characteristics through numerous passages, including passage 10 and passage 13 (Figure S1a). Additionally, iPSCs‐MSCs exhibited a higher expression of MSC‐positive surface markers (Figure S1b).

Intriguingly, we were able to observe a condensed MSC mass through the generation of teratomas using another iPSC cell line at 10 weeks (Figure S2a). Cells isolated from this condensed MSC mass demonstrated a co‐expression rate of less than 1% for MSC‐negative markers, such as CD34 and CD45, while also displaying a co‐expression rate of over 98% for MSC‐positive markers, including CD73, CD90, and CD105 (Figure S2b).

### 2.3 In vitro multi‐lineage differentiation potential of iPSCs‐MSCs

**FIGURE 4:**
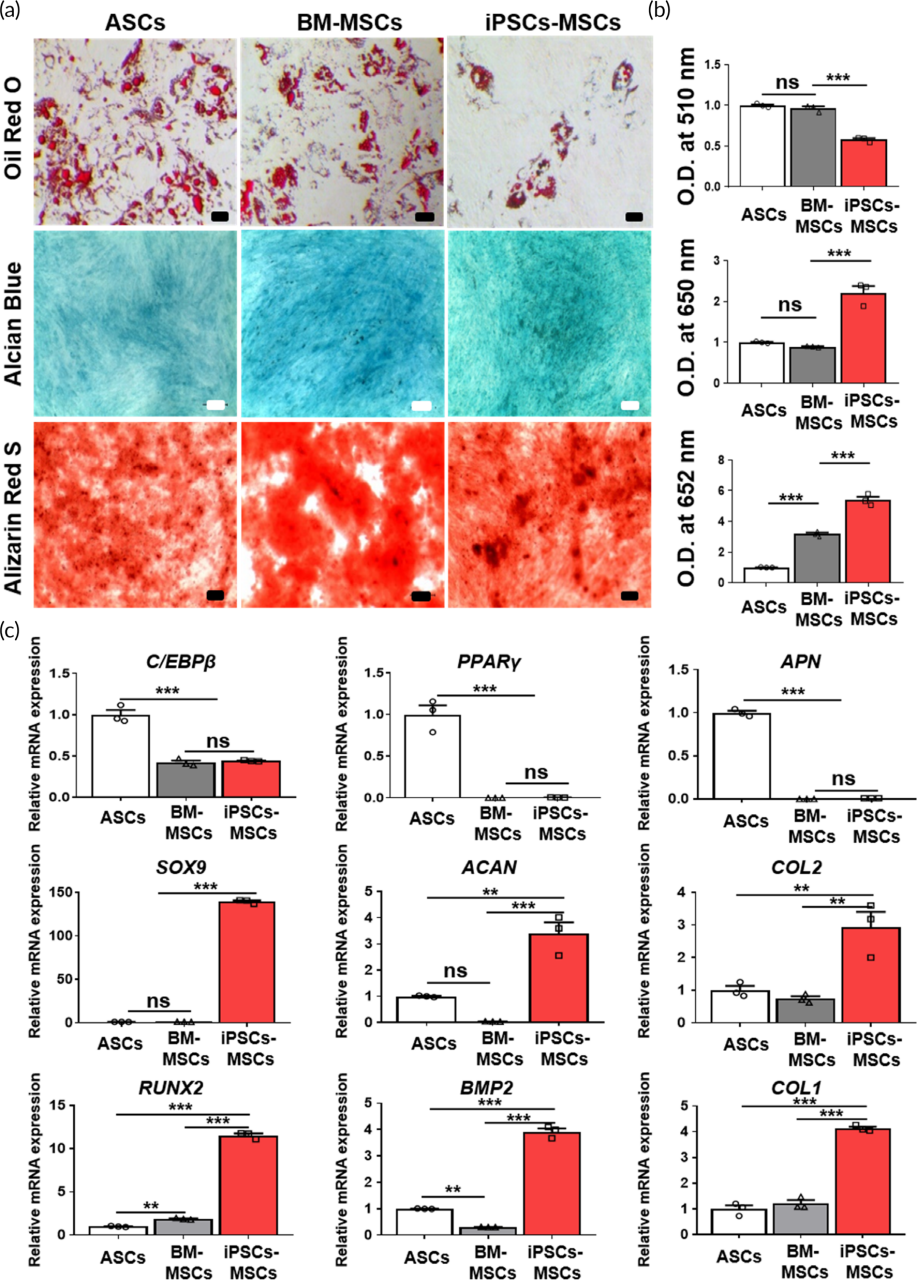
Confirmation of the in vitro multi‐lineage differentiation of iPSCs‐MSCs. (a) Representative image of differentiation potential evaluation through Oil Red O (adipogenic), Alcian blue (chondrogenic), and Alizarin Red S staining (osteogenic) and (b) the quantitative absorbance value. Scale bar, 100 μm. The data are presented as the means ± s.d. (*n* = 3, biologically independent samples). *p < 0.05, **p < 0.01, ***p < 0.001, ns: no significance; based on one‐way ANOVA followed by Tukey's post hoc test. (c) Real‐time PCR analyses were conducted to assess the differences in the mRNA expression of adipogenic markers (*C/EBPβ*, *PPARγ, APN*), chondrogenic markers (*SOX9, ACAN, COL2*), and osteogenic markers (*RUNX2, BMP2, COL1*) between ASCs, BM‐MSCs, and iPSCs‐MSCs. The data are presented as the means ± s.d. (*n* = 3, biologically independent samples). *p < 0.05, **p < 0.01, ***p < 0.001, ns: no significance; based on one‐way ANOVA followed by Tukey's post hoc test.

In this study, we conducted a straightforward evaluation of the in vitro multi‐lineage differentiation potential of iPSCs‐MSCs by comparing them to ASCs and BM‐MSCs. We employed Oil Red O staining for adipogenesis, Alcian Blue staining for chondrogenesis, and Alizarin Red S staining for osteogenesis (Figure 4a), and quantified the results using absorbance measurements after the extraction of the stained dyes (Figure 4b). The quantitative data regarding the adipogenic differentiation properties of iPSCs‐MSCs were found to be lower than those of ASCs and BM‐MSCs. In contrast, iPSCs‐MSCs stained with Alcian Blue and Alizarin Red S exhibited higher values compared to ASCs and BM‐MSCs. To gain further insights into the gene expression patterns of these cells, iPSCs‐MSCs, ASCs, and BM‐MSCs were analyzed using qPCR (as shown in Figure 4c). Adipogenic markers such as CCAAT enhancer binding protein (*C/EBPβ*), peroxisome proliferator‐activated receptor gamma (*PPARγ*), and adiponectin (*APN*) exhibited lower expression in both BM‐MSCs and iPSCs‐MSCs when compared to ASCs. Furthermore, chondrogenic markers such as collagen type 2 (*COL2*), SRY‐Box transcription factor 9 (*SOX9*), and aggrecan (*ACAN*), as well as osteogenic markers such as collagen type 1 (*COL1*), runt‐related transcription factor 2 (*RUNX2*), and bone morphogenetic protein 2 (*BMP2*), were expressed at higher levels in iPSCs‐MSCs in contrast to ASCs and BM‐MSCs.

To address our concern that iPSCs‐MSCs might resemble chondrocytes, we sought to confirm the distinctions between iPSCs‐MSCs and chondrocytes. At the gene level, we found no significant differences in *SOX9* expression. However, iPSCs‐MSCs exhibited lower levels of *ACAN* and *COL2* expression when compared to chondrocytes, while demonstrating higher expression levels of *COL10* (Figure S3).

### 2.4 Gene profiling of iPSCs‐MSCs compared with iPSCs and BM‐MSCs

**FIGURE 5:**
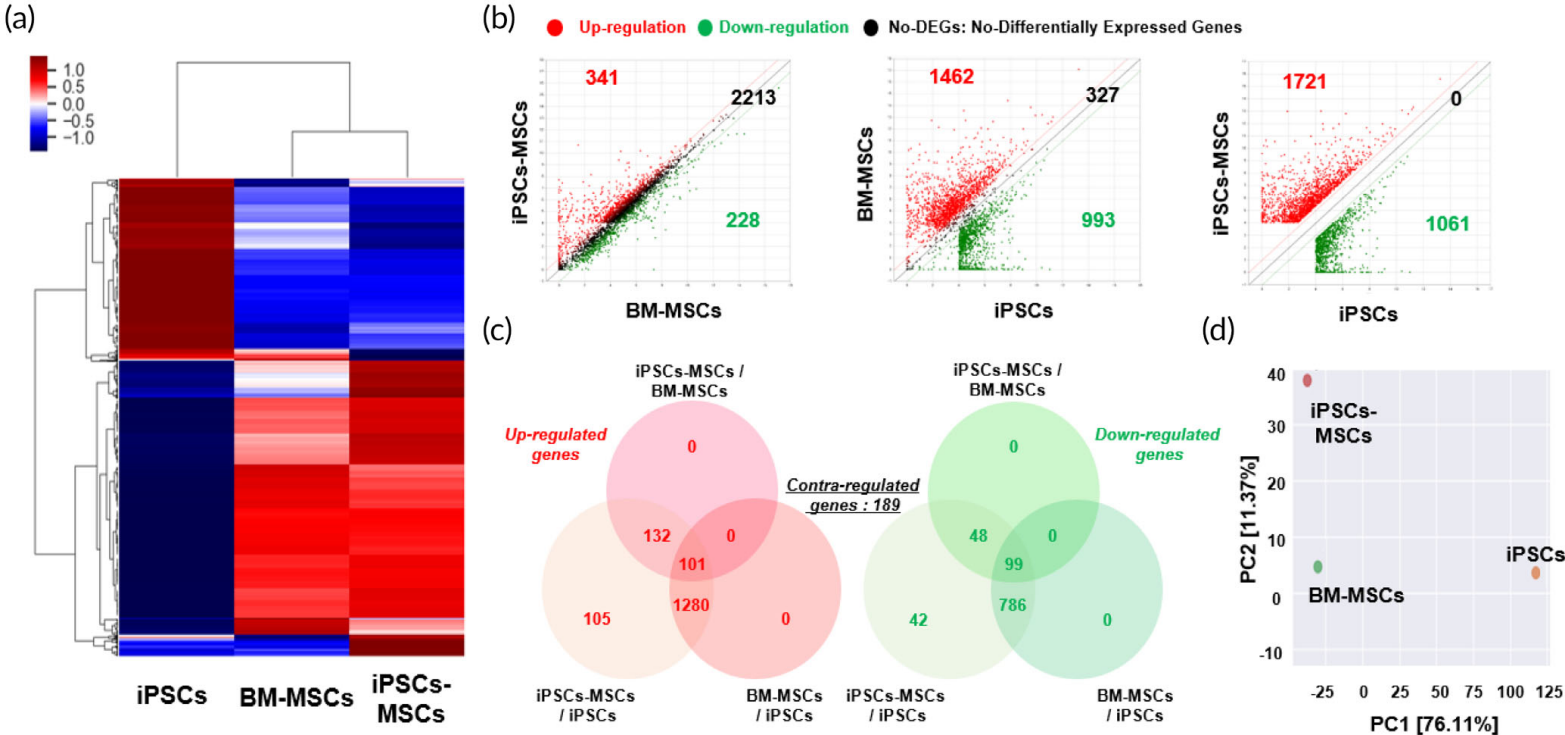
Global gene expression profile showing the biological comparison between iPSCs, BM‐MSCs, and iPSCs‐MSCs. (a) Heatmap showing the variations in gene expression patterns between iPSCs, BM‐MSCs, and iPSCs‐MSCs. (b), (c) Scatter plot and Venn diagram showing upregulated and downregulated genes between different groups (iPSCs‐MSCs vs BM‐MSCs, BM‐MSCs vs. iPSCs, and iPSCs‐MSCs vs. iPSCs). (d) Principal component analysis showing similarities between iPSCs, BM‐MSCs, and iPSCs‐MSCs.

To confirm the similarities and differences in the transcriptome profiles of human iPSCs, BM‐MSCs, and iPSCs‐MSCs, we conducted a comprehensive analysis of their transcriptomes through mRNA sequencing.^39^ Differential expression analysis revealed 2782 genes with significant modulation in iPSCs‐MSCs compared to BM‐MSCs, BM‐MSCs compared to iPSCs, and iPSCs‐MSCs compared to iPSCs (fold difference ≥6 and p‐adj <0.05). A heatmap was generated to visualize the gene expression signatures of these 2782 modulated genes in iPSCs‐MSCs, indicating that they closely resembled the expression patterns of BM‐MSCs rather than iPSCs. As anticipated, the expression patterns of the entire transcriptome in iPSCs‐MSCs closely mirrored those of BM‐MSCs (Figure 5a). Scatter plot analyses further highlighted the differential gene expression patterns. Specifically, 2074, 2668, and 947 genes were upregulated in iPSCs‐MSCs/BM‐MSCs, BM‐MSCs/iPSCs, and iPSCs‐MSCs/iPSCs, respectively, while 228, 993, and 1061 genes were downregulated in these respective comparisons (Figure 5b). The total number of differentially expressed genes, including both upregulated and downregulated genes, was substantially lower between iPSCs‐MSCs and BM‐MSCs compared to the differences observed between iPSCs‐MSCs and iPSCs. Venn diagram analysis highlighted the distinctions in regulated genes among these comparisons (Figure 5c). Interestingly, 105 genes were upregulated and 42 were downregulated in iPSCs‐MSCs compared to iPSCs, whereas 189 genes exhibited contraregulation. Furthermore, the genetic relationships between iPSCs, BM‐MSCs, and iPSCs‐MSCs were visualized by generating a principal component analysis (PCA) plot. Each group was distinctly plotted, with iPSCs, BM‐MSCs, and iPSCs‐MSCs forming separate clusters. Notably, iPSCs‐MSCs and BM‐MSCs demonstrated a closer relationship, explaining 76.11% of the variance (PCA component 1), whereas the separation between iPSCs‐MSCs and BM‐MSCs accounted for 11.37% of the variance (PCA component 2). This observation suggests that iPSCs‐MSCs share more genetic characteristics with BM‐MSCs than with iPSCs. In summary, the findings indicate that the global transcriptome and lineage signature genes in iPSCs‐MSCs are not significantly different from those in BM‐MSCs, but they are distinctly different from iPSCs.

### 2.5 Therapeutic potential of iPSCs‐MSCs in osteochondral defect regeneration

**FIGURE 6:**
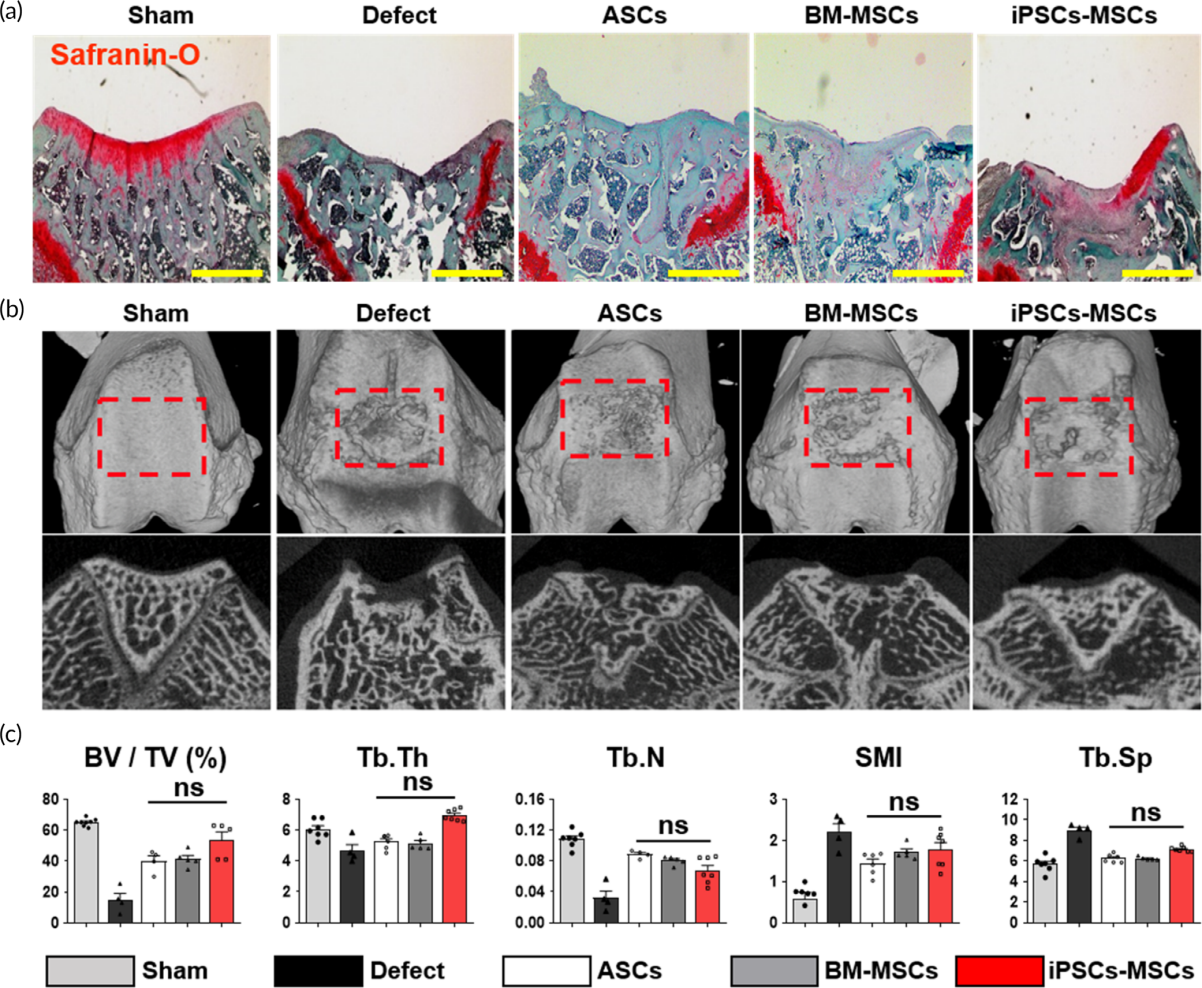
Therapeutic potential of iPSCs‐MSCs in osteochondral defect model. (a) Confirmation of chondrogenesis potential of iPSCs‐MSCs compared with ASCs and BM‐MSCs through Safranin‐O staining. Scale bar, 500 μm. (b) Three‐dimensional (3D) reconstructed image and cross‐sectional image of micro‐CT at 8 weeks. The regions of interest (ROI) indicated with the red squares were analyzed via bone histomorphometry (*n* = 6, animal per sham; *n* = 5 animal per groups). (c) The analyzed ROI data are expressed with the following parameters: percent bone volume (BV/TV, %), trabecular thickness (Tb.Th, mm), trabecular number (Tb.N, 1/mm), structure model index (SMI), and trabecular separation (Tb.Sp, mm). Data are presented as the means ± s.d. (*n* = 6 animal per sham; *n* = 4–5 animal per groups). *p < 0.05, **p < 0.01, ***p < 0.001, ns: no significance; based on one‐way ANOVA followed by Tukey's post hoc test.

Next, to assess potential therapeutic improvements, we transplanted ASCs, BM‐MSCs, and iPSCs‐MSCs, along with 2% hyaluronic acid, into an osteochondral defect site in rats using a rat osteochondral defect model (Figure 6). Male rats were exclusively selected for this in vivo study because the iPSCs‐MSCs were derived from male iPSCs. Eight weeks after MSC implantation, Safranin‐O staining was conducted to quantify cartilage regeneration, revealing that iPSCs‐MSCs exhibited a cartilage regeneration potential similar to that of ASCs and BM‐MSCs (see Figure 6a). Additionally, 3D microcomputed tomography (micro‐CT) analysis was employed to assess bone regeneration (refer to Figure 6b). Several indices, including the bone volume over total volume ratio (BV/TV, %), trabecular thickness (Tb.Th), trabecular number (Tb.N), structure model index (SMI), and trabecular spacing (Tb.Sp), were investigated (as depicted in Figure 6c). As mentioned earlier, many parameters showed no significant differences among the groups treated with ASCs, BM‐MSCs, and iPSCs‐MSCs. Our results thus confirmed that iPSCs‐MSCs exhibit effective in vivo tissue formation potential on par with ASCs and BM‐MSCs.

Residual or endogenous safety concerns are crucial issues that limit the utilization of teratoma tissues. Immunohistochemistry was conducted using human nuclei and mouse nuclei antigen staining, revealing that there was no interpenetration of mouse cells into the condensed MSCs mass (Figure S4a). Furthermore, the possibility of mycoplasma contamination was ruled out using a MycoAlert™ mycoplasma detection kit, which confirmed the absence of mycoplasma contamination in iPSCs‐MSCs (Figure S4b). Finally, karyotype analysis of iPSCs‐MSCs demonstrated that they exhibited a normal chromosome number (46, XY, consistent with the normal chromosome count of iPSCs) and phenotype (Figure S4c).

## 3 DISCUSSION

To utilize MSCs as a cell source for stem cell therapeutics, numerous studies have sought to identify new sources of MSCs from various tissues within the human body and from PSCs.^10^, ^11^, ^40^, ^41^, ^42^ However, the availability of MSCs derived from somatic tissues is limited due to the rarity of their origin and the need for diverse donors. Furthermore, PSC‐derived MSCs often require complex treatments involving growth factors and cytokines over an extended culture period, as they are derived in in vitro culture conditions.

Teratomas are benign tumors that can be differentiated from metastatic malignant cancers.^43^ These tumors can consist of mature or immature tissues originating from all three germ layers, including the endoderm, mesoderm, and ectoderm. When PSCs are injected, they spontaneously differentiate into cells from all three germ layers, including mesodermal cells. Recent studies have highlighted the importance of Matrigel, a mixture of mouse tumor extracellular matrix proteins, in the generation and culture of organoid tissues.^44^ Therefore, the development of three germ layer‐related tissues and cells within teratomas can be comparable to or even superior to those grown in Matrigel. Based on this premise, we hypothesized that mesodermal tissue from teratomas could serve as a source of MSCs. Consequently, we aimed to establish a novel method for obtaining human PSC‐derived MSCs through the formation of teratomas. Notably, the subcutaneous space provides a microenvironment conducive to the spontaneous differentiation of PSCs,^45^ leading to the development of teratomas with tissues from all three germ layers, a finding supported by histological analysis. Our observations revealed that the weight and number of condensed MSC masses generated from teratomas varied over time. These results suggested that the duration of teratoma development depended on factors such as the viability of the transplanted iPSC lines. Moreover, the condensed MSC mass, being part of the mesodermal tissue developed within teratomas, can be considered as a potential source of MSCs.^46^ Therefore, our findings suggested that the optimal time point for efficiently isolating the condensed MSC mass is 10 weeks after iPSC transplantation.

Unexpectedly, iPSCs‐MSCs exhibited higher expression of MSC‐positive markers and lower expression of pluripotent and MSC‐negative markers, similar to ASCs and BM‐MSCs, suggesting that iPSCs‐MSCs possessed MSC‐like characteristics. Although the mRNA expression level of *CD105* in iPSCs‐MSCs was lower than that in ASCs and BM‐MSCs, a significant difference was observed when compared to iPSCs. This was corroborated through immunophenotype analysis conducted via flow cytometry. Furthermore, iPSCs‐MSCs exhibited low expression of CD11b, a positive marker of leukocytes^47^ and CD34, a positive marker of endothelial cells^48^ and embryonic fibroblast cells.^49^ These results suggest that iPSCs‐MSCs do not exhibit characteristics of leukocytes, endothelial cells, or embryonic fibroblast cells. Additionally, iPSCs‐MSCs can be considered a valuable source of MSCs due to their increased proliferative capacity, allowing for the generation of a substantial number of cells,^50^ similar to our iPSCs‐MSCs.

Our findings suggest that iPSCs‐MSCs differentiate into cells with MSC‐like characteristics and exhibit superior proliferative capacity compared to both ASCs and BM‐MSCs, even at high passages. Furthermore, we validated the isolation potential of CD45^−^CD90^+^CD105^+^ cells as representative MSCs surface phenotypes from condensed MSCs mass using another iPSC cell line‐generated teratomas. This indicates that condensed MSC masses could be utilized with various types of PSCs for MSC isolation. These findings highlight the utility of human iPSC‐derived teratomas as a valuable microenvironment for generating condensed MSC masses to isolate human MSCs.

Verification of the differentiation capability of iPSCs‐MSCs at the mRNA level demonstrated their in vitro multi‐lineage differentiation potential, with higher chondrogenic and osteogenic properties compared to ASCs and BM‐MSCs, although adipogenic properties were not as pronounced. This suggests that even MSCs generated from iPSCs may exhibit diverse differentiation properties influenced by donor variations. These findings align with the work of Eto et al., who reported varying therapeutic potentials of iPSC‐derived MSCs in different mouse disease models.^51^ Interestingly, if these condensed MSC masses were generated through the endochondral ossification process,^37^ the predominant cell population in iPSCs‐MSCs would exhibit chondrocyte characteristics. Their appearance also resembled that of cartilage tissue during the teratoma development process from iPSCs. Therefore, it was essential to compare the general characteristics of iPSCs‐MSCs with human normal chondrocytes (Chon) at the gene level. This analysis revealed that the intracellular molecules in iPSCs‐MSCs were distinct from those in human chondrocytes. Furthermore, *COL10*, an important marker for MSCs in the endochondral ossification pathway, exhibited higher expression levels in iPSCs‐MSCs compared to chondrocytes.^52^ Collectively, our findings demonstrated that iPSCs‐MSCs were comparable to ASCs and BM‐MSCs, displayed in vitro multi‐lineage differentiation, and exhibited superior therapeutic potential for enhancing chondrogenic and osteogenic differentiation properties.

To confirm the clinical applicability of iPSCs‐MSCs generated via teratomas, we verified the absence of mouse nuclei and mycoplasma contamination, as well as the presence of a normal karyotype. Nevertheless, it is worth noting that these safety‐related confirmations are insufficient to rule out interspecies contamination, as we did not evaluate the presence of mouse proteins in iPSCs‐MSCs generated from teratomas. Notably, iPSC‐generated teratomas could develop through exposure to mouse proteins circulating in the mouse blood during teratoma development.

## 4 CONCLUSIONS

To overcome the limitations associated with conventional methods for deriving and differentiating MSCs from PSCs, we proposed a novel approach utilizing teratomas as a potential MSC source. To demonstrate the in vivo generation of MSCs, we injected iPSCs into the subcutaneous space to induce teratoma formation. Within these teratomas, we observed a condensed mass of MSCs, which we subsequently examined. Our findings indicated that this condensed MSC mass could serve as a source of iPSC‐derived MSCs. We also sought to optimize the conditions for isolating MSCs from teratomas and assess their proliferative capacity over numerous passages. This observation was further validated using another iPSC cell line. Furthermore, our findings demonstrated that iPSC‐derived MSCs exhibited multi‐lineage differentiation properties and therapeutic potential in the context of bone and cartilage tissue formation, all while avoiding severe contamination. As a result, our study was the first to isolate human MSCs from teratomas via human iPSCs. Our innovative method outperforms conventional approaches by eliminating the need for (1) sorting MSCs with flow cytometry, (2) prolonged in vitro cultivation, and (3) employing complex combinations of growth factors and cytokines for treatment. Most notably, our innovative approach allows for the in vivo isolation of MSCs from teratomas, as opposed to the traditional in vitro culture environment. This brings us closer to the derivation and growth of naïve MSCs in the context of human embryo development. Thus, we are confident that MSCs derived within the teratoma environment will surpass those derived in conventional in vitro culture settings.

## 5 MATERIALS AND METHODS

### 5.1 iPSCs culture

The human iPSCs line hFSiPS1, which was derived from male dermal fibroblasts,^53^ was acquired from the National Stem Cell Bank of Korea. The iPSCs were routinely maintained for 5–6 days on a dish coated with Matrigel (Corning) using Essential 8 (E8) feeder‐free medium (Invitrogen). To prepare the Matrigel‐coated dish, Matrigel was diluted in Dulbecco's Modified Eagle's Medium (DMEM)/F12 medium (Invitrogen) to create a 1% Matrigel solution. The dish was then coated with this 1% Matrigel solution at 4°C for 18 h. Before conducting the experiments, the Matrigel‐coated dish was thoroughly washed with PBS. Next, the iPSCs were dissociated using 5 mM EDTA (Invitrogen) at 37°C for 4 min. Following the EDTA treatment, iPSC colonies were dissociated into clumps within the culture medium. These clumps were then seeded onto the newly Matrigel‐coated dish, which was supplemented with E8 medium and 3 μM y‐27632 (Tocris). The culture medium was refreshed daily, starting from the second day of seeding.

### 5.2 Teratoma formation assay

These in vivo experiments were approved by the Dongguk University IACUC‐2019‐047‐2, in accordance with ARRIVE guidelines (https://arriveguidelines.org/arrive-guidelines). For the experiments, iPSCs were injected into the subcutaneous space of randomly selected 7‐week‐old immunodeficient female nude mice. Briefly, when the iPSCs reached approximately 80%–95% confluency, they were washed with PBS and then dissociated using 5 mM EDTA. The iPSCs were subsequently collected in PBS and centrifuged at 400 x *g* for 3 min. After removing the supernatant, the iPSCs were gently resuspended in Matrigel on ice to achieve a concentration of 2 × 10^7^ cells per milliliter. Afterward, approximately 2 × 10^6^ cells in 100 μL of Matrigel were injected into a single subcutaneous site, as previously described.^45^ Teratomas were then surgically harvested in the subsequent weeks following the injection.

### 5.3 Collection of condensed MSCs mass and isolation of iPSCs‐MSCs

At specific time points following the transplantation of iPSCs, teratomas were harvested from the subcutaneous space, and condensed masses of MSCs were extracted from within the teratomas using a surgical procedure. The identification of these condensed MSC masses was facilitated by the presence of small, cartilage‐like tissues, with diameters typically ranging from 3 to 5 mm. These tissues displayed a rounder and semi‐solid appearance compared to other tissues within the teratomas. The isolated condensed MSC masses were washed twice with a solution of 2% antibiotics in PBS, after which they were finely chopped using a surgical blade. Afterward, the samples were digested for 12 h in a 0.5 mg/mL collagenase solution at 37°C. Following digestion, the dissociated condensed MSC masses were treated with MSC medium composed of Dulbecco's Modified Eagle Medium (DMEM/Low glucose media), 10% FBS, and 1% Penicillin/Streptomycin (P/S, Gibco), after which they were filtered through a 40‐μm strainer. The filtered mixture was centrifuged at 1000 rpm for 10 minutes and resuspended in MSC medium three times. The cell pellet was dissociated using MSC medium and then plated onto a culture dish. The MSC medium was refreshed after 24 h. iPSCs‐MSCs were passaged every 7 days using 0.25% trypsin‐EDTA (Highclone), and the MSC medium was changed every 2 days.

### 5.4 H&E staining

The samples were decalcified with 5% nitric acid for 24 h at room temperature, followed by dehydration using a series of graded ethanol and xylene. Subsequently, the samples were embedded in paraffin. Sections of these samples were then deparaffinized with xylene, dehydrated with ethanol, and subjected to hematoxylin and eosin staining (H&E).

### 5.5 Cell proliferation capacity

The cells (passage 2) were initially seeded into 6‐well plates with a density of 50,000 cells per well and cultured using MSC medium. For subculturing, cells were rinsed with PBS and detached using 0.25% trypsin‐EDTA every 4 days. Subsequently, the cell count was recorded using LUNA‐II™ until passage 13, at which point both ASCs and BM‐MSCs exhibited apoptosis. The cell cycle was assessed using a propidium iodide staining protocol (PI staining). The harvested cells were washed with PBS and then fixed with cold 70% ethanol at 4°C for 18 h. After fixation, the cells were rinsed with PBS twice, and 50 μL of RNase A (Invitrogen) was introduced. Cells were then treated with a total of 200 μL of propidium iodide solution (Sigma) and incubated for 15 minutes in the dark. The fluorescence resulting from the PI staining was analyzed using a BD Accuri C6 flow cytometer (Becton Dickinson, USA).

### 5.6 RNA isolation and quantitative real‐time polymerase chain reaction (qRT‐PCR)

The total RNA from the cells was extracted using Trizol reagent from Invitrogen. For quantitative real‐time polymerase chain reaction (qPCR), all the cells were cultured in 6‐well plates. In each well, the cells were washed with 1 mL of PBS and then treated with 500 μL of Trizol reagent. The cells were then incubated at room temperature for 5 min and then scraped to collect the reagent. This collected reagent was mixed with 200 μL of chloroform and incubated on ice for 10 min. The mixture was subsequently centrifuged at 13,000 rpm for 15 min, and the aqueous supernatant was gently collected. The collected supernatant was combined with an equal volume of isopropanol and incubated on ice for an additional 10 min. The RNA sample was then centrifuged at 13,000 rpm for 15 min, and the supernatant was discarded. The RNA pellet was washed with 75% ethanol and allowed to air dry. The resulting transparent RNA pellet was diluted in nuclease‐free water. Afterward, the RNA concentration was determined using a Cytation 3 instrument, and 1 μg of RNA was used for cDNA synthesis. The cDNA was synthesized using the PrimeScript RT Reagent kit (TAKARA, Japan). qPCR analyses were carried out using the Power SYBR Green PCR Master mix (ThermoFisher Scientific, USA) and the ΔΔ*CT* value was calculated using a Step‐One perfect qPCR machine, also from ThermoFisher Scientific. The primer sequences employed for the qPCR analysis are summarized in Table 1. GAPDH was used as a reference gene for normalizing sample amplifications.

**TABLE 1:** Primer sequences for qRT‐PCR.

Gene	Forward primer (5′‐3′)	Reverse primer (5′‐3′)
*OCT4*	CCT TCG CAA GCC CTC ATT TCA	AAA TCC GAA GCC AGG TGT CC
*SOX2*	GGA TAA GTA CAC GCT GCC CG	ATG TGC GCG TAA CTG TCC AT
*CDH1*	CCC GGG ACA ACG TTT ATT AC	GCT GGC TCA AGT CAA AGT CC
*CD73*	CAG TAC CAG GGC ACT ATC TGG	AGT GGC CCC TTT GCT TTA AT
*CD105*	CCA CTA GCC AGG TCT CGA AG	GAT GCA GGA AGA CAC TGC TG
*C/EBPβ*	GCA AGA GCC GCG ACA AG	GGC TCG GGC AGC TGC TT
*PPARγ*	GAT ACA CTG TCT GCA AAC ATA TCA CAA	CCA CGG AGC TGA TCC CAA
*APN*	TTC CAT ACC AGA GGG GCT CA	CCC TTG AGT CGT GGT TTC CT
*RUNX2*	CAG ACC AGC AGC ACT CCA TA	CAG CGT CAA CAC CAT CAT TC
*BMP2*	CCA CTC TCC AGG CGT ACC T	CGG ACT GCG GTC TCC TAA
*COL1*	CCC CTG GAA AGA ATG GAG ATG	TCC AAA CCA CTG AAA CCT CTG
*GAPDH*	ACA TCG CTC AGA CAC CAT G	TGT AGT TGA GGT CAA TGA AGG G

### 5.7 Flow cytometry analysis

To assess the immunophenotype of the cells, we first washed the cells with PBS and detached them using 0.25% trypsin‐EDTA. All of the cells were then fixed with 4% paraformaldehyde at 4°C for 18 h, after which the paraformaldehyde was discarded after centrifugation at 400 × *g* for 3 min. The fixed samples were then washed three times with a solution of 2% FBS in PBS. Flow cytometry analyses were conducted with the following primary antibodies: OCT4‐FITC (Biolegend), SOX2‐FITC (Biolegend), CD90‐APC (Biolegend), CD73‐PE (Biolegend), CD105‐PE (Biolegend), CD11b‐PE (Biolegend), CD34‐PerCP (Biolegend), CD45‐PE (Biolegend), and HLA‐DR‐APC (Biolegend). These antibodies were used at a 1:100 dilution and incubated at 4°C for 1 h in a dark environment. Afterward, the cells were washed three times with a 2% FBS in PBS solution, and fluorescence signals were detected using a BD Accuri C6 flow cytometer (BD Science, USA).

### 5.8 Confirmation of multi‐lineage differentiation of MSCs

To confirm the multipotent properties of iPSCs‐MSCs, we induced adipogenic, chondrogenic, and osteogenic differentiation in iPSCs‐MSCs, ASCs, and BM‐MSCs using a chemically defined medium at passage 3. Adipogenic and chondrogenic differentiation were induced for 2 weeks, while osteogenic differentiation was induced for 3 weeks. We changed the medium every 2 days. The adipogenic differentiation medium was prepared using DMEM/High glucose media (High media) containing 10% FBS, 1% P/S, 10 μg/mL insulin (Sigma), 500 μM 3‐isobutyl‐1‐methyl xanthine (IBMX, Sigma), 200 μM indomethacin (Sigma), and 1 μM dexamethasone (Sigma). The chondrogenic differentiation medium was composed of DMEM/High media with 10% FBS, 1% P/S, 1% Insulin‐Transferrin‐Selenium‐A (Gibco BRL), 50 μg/mL ascorbic acid (Sigma), 100 nM dexamethasone, and 10 ng/mL transforming growth factor β1 (TGF‐β1, Peprotech). The osteogenic differentiation medium consisted of DMEM/High media with 10% FBS, 1% P/S, 10 mM glycerol‐2‐phosphate (Sigma), 50 μg/mL ascorbic acid, 100 nM dexamethasone, and 1% GlutaMAX (Gibco).

### 5.9 Evaluation of multipotency using histological analysis with an in vivo study

All in vivo experiments involved in this study were approved by the Dongguk University IACUC‐2019‐047‐3, which aligns with the ARRIVE guidelines (https://arriveguidelines.org/arrive-guidelines). An osteochondral defect model was created to confirm the tissue regeneration potential of iPSCs‐MSCs. Prior to initiating the in vivo study, power analyses were performed to minimize the number of animals required for our experiments. Initially, 7‐week‐old male rats were randomly divided into 5 groups, with 6 animals in the sham group and 5 animals in each experimental group. Subchondral defects were created in their knees, with dimensions of 2 mm in diameter and 2 mm in depth. Subsequently, 1 × 10^6^ MSCs were implanted along with 2% hyaluronic acid (Sigma). After 8 weeks of treatment, the rats were euthanized using carbon dioxide, and their knee joints were fixed with 4% paraformaldehyde for 3 days. The samples were decalcified using 5% nitric acid for 24 h at room temperature. Following decalcification, the samples were dehydrated with graded ethanol and xylene before being embedded in paraffin. Sections of the samples were then deparaffinized with xylene, dehydrated with ethanol, and stained with Safranin‐O and Nuclear Fast Green.

### 5.10 Library preparation and RNA sequencing

A library was constructed using the SENSE mRNA‐Seq Library Prep Kit from Lexogen, following the manufacturer's instructions. Briefly, 2 μg of total RNA was prepared and incubated with magnetic beads decorated with oligo‐dT, leading to the removal of all RNAs except mRNAs. The library preparation began with the random hybridization of starter/stopper heterodimers to the poly(A) RNA that remained bound to the magnetic beads. These starter/stopper heterodimers contained Illumina‐compatible linker sequences. A single‐tube reverse transcription and ligation reaction extended the starter to the next hybridized heterodimer, where the newly synthesized cDNA insert was ligated to the stopper. Second‐strand synthesis was performed to release the library from the beads, and the library was then amplified, introducing barcodes during the amplification process. High‐throughput sequencing (paired‐end 100 sequencing) was performed using an Illumina HiSeq 2000 sequencer. RNA‐Seq reads were mapped using the TopHat software to generate an alignment file, which was subsequently used for transcript assembly, abundance estimation, and the detection of a differential gene or isoform expression using Cufflinks. Gene classification was based on searches conducted in the BioCarta (http://www.biocarta.com/), GenMAPP (http://www.genmapp.org/), DAVID (http://david.abcc.ncifcrf.gov/), and Medline databases (http://www.ncbi.nlm.nih.gov/). Library preparation and RNA sequencing were performed using the NGS services provided by Ebiogen Inc. (Seoul, South Korea).

### 5.11 Statistical analysis

All data are expressed as means ± standard deviation (s.d.). The statistical significance of the data was assessed using one‐way analysis of variance followed by Tukey's post hoc test, which was employed for comparisons among more than two experimental groups with a single varying parameter. Statistical significance was denoted using asterisks [*(*p* < 0.05), ** (*p* < 0.01), *** (*p* < 0.001)]. Independent biological replicates were utilized to determine the n values. The calculations for all data were performed using the GraphPad Prism (Version 5.00) software.

## AUTHOR CONTRIBUTIONS

**Jiseong Kim:** Data curation (equal); investigation (equal); methodology (equal); writing – original draft (equal). **Jin‐Su Kim:** Data curation (equal); formal analysis (equal); investigation (equal); methodology (equal). **Dohyun Kim:** Formal analysis (supporting); methodology (supporting). **Alvin Bacero Bello:** Data curation (supporting); formal analysis (supporting). **Byoung Ju Kim:** Data curation (supporting); formal analysis (supporting); methodology (supporting). **Byung‐Hyun Cha:** Conceptualization (equal); data curation (equal); investigation (equal); methodology (equal); supervision (equal); writing – review and editing (equal). **Soo‐Hong Lee:** Conceptualization (equal); funding acquisition (equal); supervision (equal); writing – review and editing (equal).

## FUNDING INFORMATION

This research was supported by the National Research Foundation of Korea (NRF) grant funded by the Korean government (MSIT) (NRF‐2019M3A9H1032376, 2022R1A2C3004850 and RS‐2023‐00208529). Additionally, this study was supported by the 2023 Research Grant from Kangwon National University (202304810001).

## CONFLICT OF INTEREST STATEMENT

The authors declare no competing interests.

### PEER REVIEW

The peer review history for this article is available at https://www.webofscience.com/api/gateway/wos/peer-review/10.1002/btm2.10629.

## Supporting information


**Data S1:** Supporting Information

## Data Availability

All data are available upon reasonable request from the corresponding author.
