# Supplementary material for: Therapeutic potential of mesenchymal stem cells from human iPSC‐derived teratomas for osteochondral defect regeneration
Source: Bioeng Transl Med. 2023 Nov 29;9(2):e10629. doi: 10.1002/btm2.10629 (PMC10905541; doi:10.1002/btm2.10629)
Supplement: Supplementary file 1 — Data S1: Supporting Information [file BTM2-9-e10629-s001.docx]

Supporting Information

**Therapeutic potential of mesenchymal stem cells from human iPSC-derived teratomas for osteochondral defect regeneration**

Jiseong Kim^1,§^, Jin-Su Kim^2, §^, Dohyun Kim^1^, Alvin Bacero Bello^1,3^, Byoung Ju Kim^,4^, Byung-Hyun Cha^5^* and Soo-Hong Lee^1^*

**Material and methods**

**Induced pluripotent stem cell (hiPSC) culture**

The human induced pluripotent stem cell line hiPSC-CMC-022, which was derived from female cord blood, was acquired from the National Stem Cell Bank of Korea. The iPSCs were routinely maintained for 5–6 days on a dish coated with Matrigel (Corning) using Essential 8 (E8) feeder-free medium (Invitrogen). To prepare the Matrigel-coated dish, Matrigel was diluted in Dulbecco’s Modified Eagle’s Medium (DMEM)/F12 medium (Invitrogen) to create a 1% Matrigel solution. The dish was then coated with this 1% Matrigel solution at 4 °C for 18 hours. Before conducting the experiments, the Matrigel-coated dish was thoroughly washed with PBS. Next, the iPSCs were dissociated using 5 mM EDTA (Invitrogen) at 37 °C for 4 minutes. Following the EDTA treatment, iPSC colonies were dissociated into clumps within the culture medium. These clumps were then seeded onto the newly Matrigel-coated dish, which was supplemented with E8 medium and 3 μM y-27632 (Tocris). The culture medium was refreshed daily, starting from the second day of seeding.

**Teratoma formation**

The *in vivo* experiments were approved by the Dongguk University IACUC-2019-047-2, in accordance with ARRIVE guidelines (https://arriveguidelines.org/arrive-guidelines). For the experiments, iPSCs were injected into the subcutaneous space of randomly selected 7-week-old immunodeficient female nude mice. Briefly, when the iPSCs reached approximately 80%–95% confluency, they were washed with PBS and then dissociated using 5 mM EDTA. The iPSCs were subsequently collected in PBS and centrifuged at 400 x *g* for 3 minutes. After removing the supernatant, the iPSCs were gently resuspended in Matrigel on ice to achieve a concentration of 2 x 10^7^ cells per milliliter. Afterward, approximately 2 × 10^6^ cells in 100 μL of Matrigel were injected into a single subcutaneous site, as previously described^55^. Teratomas were then surgically harvested in the subsequent weeks following the injection.

**Safety confirmation of heterogenic contamination of iPSCs-MSCs**

Samples for immunofluorescence were washed with PBS and fixed in 4% paraformaldehyde at 4°C for 18 hours. Fixed tissues were dehydrated with graded EtOH, treated with xylene 3 times, and embedded in paraffin. Paraffin samples were cleared with xylene 3 times and hydrated. Hydrated samples were treated with 3% H_2_O_2_ in MeOH for 10 minutes and pepsin for 10 minutes to induce antigen retrieval. At each step, the samples were washed with PBS 3 times and blocked with blocking solution in a GBI kit (GBI Lab, USA) for 1 hr. Anti-human nuclei antigen antibody (Chemicon, USA) and anti-mouse nuclei antigen antibody (Clone: 28-14-8, OriGene, USA) were used at 1:50 at 4°C for 18 hours. Then, the samples were washed with 0.05% tween 20 and 3 times with PBS (PBS-T), and secondary antibodies conjugated with Alexa 488 or Texas red (Invitrogen USA) were treated 1:500 at room temperature for 2 hours in a dark state. Samples were washed 3 times with PBS-T and stained with 4′,6-diamidino- 2-phenylindole dihydrochloride (DAPI, Sigma) for 10 minutes at RT. The fluorescence was detected using an IX70 Olympus fluorescence microscope (Olympus, Japan). Karyotype profiling was performed by Gendix (Seoul, South Korea).

**RNA isolation and quantitative real-time polymerase chain reaction (qRT-PCR)**

The total RNA from the cells was extracted using Trizol reagent from Invitrogen. For quantitative real-time polymerase chain reaction (qPCR), all the cells were cultured in 6-well plates. In each well, the cells were washed with 1 mL of PBS and then treated with 500 μL of Trizol reagent. The cells were then incubated at room temperature for 5 minutes and then scraped to collect the reagent. This collected reagent was mixed with 200 μL of chloroform and incubated on ice for 10 minutes. The mixture was subsequently centrifuged at 13,000 rpm for 15 minutes, and the aqueous supernatant was gently collected. The collected supernatant was combined with an equal volume of isopropanol and incubated on ice for an additional 10 minutes. The RNA sample was then centrifuged at 13,000 rpm for 15 minutes, and the supernatant was discarded. The RNA pellet was washed with 75% ethanol and allowed to air dry. The resulting transparent RNA pellet was diluted in nuclease-free water. Afterward, the RNA concentration was determined using a Cytation 3 instrument, and 1 μg of RNA was used for cDNA synthesis. The cDNA was synthesized using the PrimeScript RT Reagent kit (TAKARA, Japan). qPCR analyses were carried out using the Power SYBR Green PCR Master mix (ThermoFisher Scientific, USA) and the ΔΔCT value was calculated using a Step-One perfect qPCR machine, also from ThermoFisher Scientific. The primer sequences employed for the qPCR analysis are summarized in Table S1. GAPDH was used as a reference gene for normalizing sample amplifications.

**Mycoplasma detection**

Mycoplasma contamination of Td-MSCs was confirmed with the MycoAlert^TM^ PLUS Mycoplasma Detection Kit (Lonza, Switzerland) as described. Briefly, 2 mL of conditioned medium of Td-MSCs was collected and centrifuged at 200 xg for 5 minutes. A total of 100 μL of fresh supernatant was collected. The fresh supernatant was mixed with MycoAlert^TM^ PLUS reagent and incubated at room temperature for 5 minutes. The luminescence of the mixture (Read A) was measured by Cytation 3. MycoAlert^TM^ PLUS substrate was added to the mixture and incubated at room temperature. The luminescence of mixture (Read B) was measured after 10 minutes, and the ratio of Read B/ Read A was calculated to confirm mycoplasma contamination.

**Cell cycle analysis**

The cell cycle was confirmed using a propidium iodide staining protocol (PI staining). The harvested cells were washed with PBS and fixed with cold 70% EtOH at 4°C for 18 hours. The fixed cells were washed with PBS 2 times, and 50 μL of RNase A (Invitrogen) was added. A total of 200 μL of propidium iodide (Sigma) solution was used to treat cells, which were then incubated for 15 minutes in the dark. The fluorescence of the PI staining was detected with BD Accuri C6 flow cytometry (Becton Dickinson, USA).

**Immunophenotype analysis**

To detect the immunophenotype of the cells, cells were washed with PBS and detached by 0.25% trypsin. All of the cells were fixed with 4% paraformaldehyde at 4°C for 18 hr. Paraformaldehyde was discarded after centrifugation at 400 xg for 3 minutes. The fixed sample was washed with 2% FBS in PBS 3 times. The following primary antibodies were used for flow cytometry: CD90-APC (Biolegend), CD73-PE (Biolegend), CD105-PE (Biolegend), CD34-PerCP (Biolgend), and CD45-PE (Biolegend). The antibodies were treated as 1:100 diluents and incubated at 4°C for 1 hours in the dark state. Cells were washed with 2% FBS in PBS solution 3 times, and the fluorescence was detected by BD Accuri C6 (BD science, USA).

**Supplementary Figure**


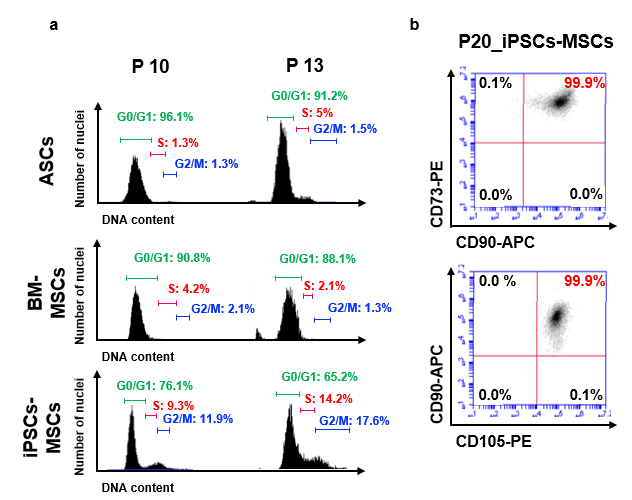


**Supplementary Fig. 1** Characterization of the cell cycle and stability in high passaged iPSCs-MSCs. **a** PI staining for cell cycle analysis of ASCs, BM-MSCs, and Td-MSCs at P10 and P13. **b** Flow cytometry analysis of MSC markers CD73, CD90, and CD105 at passage 20 of Td-MSCs.


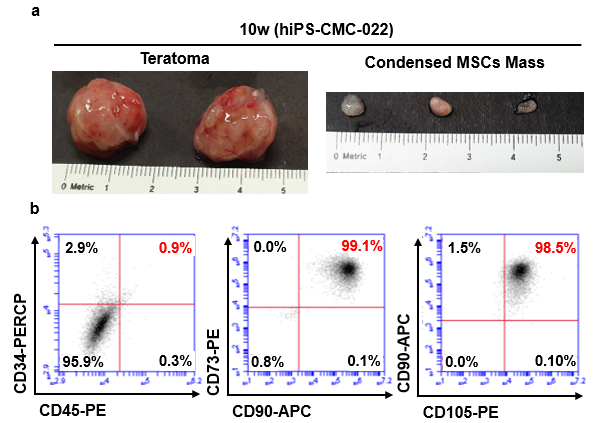


**Supplementary Fig. 2** Characterization of iPSCs-MSCs isolated from white tissue using another iPSC line. **a** Macroscopic images showing establishment of teratomas and white tissue by another iPSC line. (n = 6, biologically independent samples). **b** Flow cytometry analysis of MSC-negative markers (CD34, and CD45) and MSC-positive markers (CD73, CD90, and CD105) of iPSCs-MSCs isolated from condensed MSCs mass.


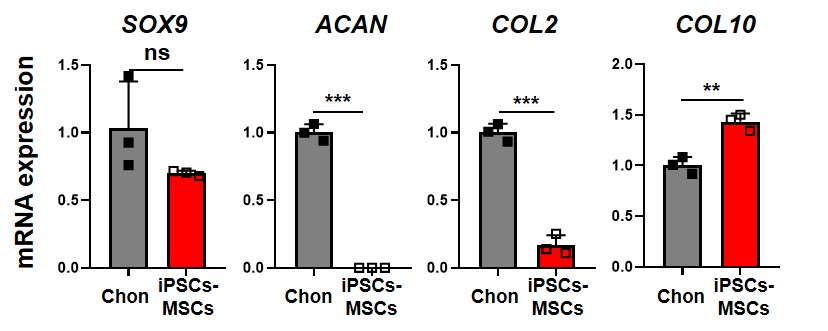


**Supplementary Fig. 3** Comparison of mRNA expression between chondrocytes and iPSCs-MSCs. qRT-PCR analysis was performed to demonstrate that iPSCs-MSCs have different characteristics compared with chondrocytes (chon). Data are presented as the means ± s.d. (n = 3, biologically independent samples). **P* < 0.05, ***P* < 0.01, ****P* < 0.001, ns: no significance; based on one-way ANOVA followed by Tukey’s post hoc test.


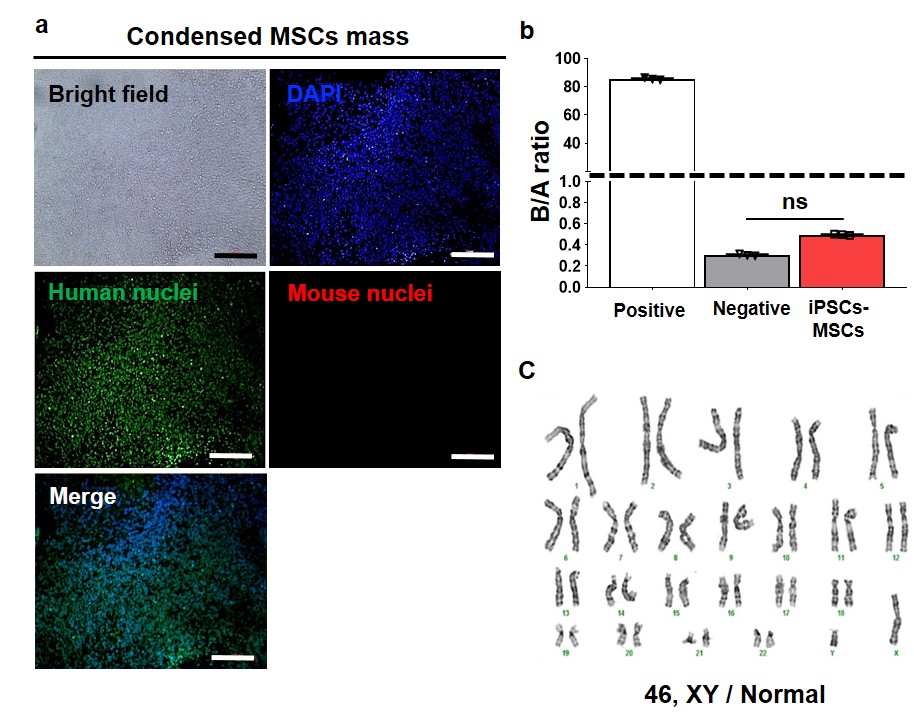


**Supplementary Fig. 4.** Safety confirmation of iPSCs-MSCs. **a** Immunofluorescence staining with mouse nuclei and human nuclei antibodies. Scale bar, 200 μm. **b** Mycoplasma contamination analysis of iPSCs-MSCs using the MycoAlert^TM^ mycoplasma detection kit. Data are presented as the means ± s.d. (n = 3, biologically independent samples). ns: no significance; based on one-way ANOVA followed by Tukey’s post hoc test. **c** Representative image of karyotype profiling of iPSCs-MSCs.

**Supplementary Table**

**Supplementary Table 1.** Primer sequences for qRT-PCR.

| **Gene** | **Forward primer (5’-3’)** | **Reverse primer (5’-3’)** |
| --- | --- | --- |
| *SOX9* | GTA CCC GCA CTT GCA CAA C | TCT CGC TCT CGT TCA GAA GTC |
| *ACAN* | GCC TGC GCT CCA ATG ACT | ATG GAA CAC GAT GCC TTT CAC |
| *COL2* | CAC GTA CAC TGC CCT GAA GGA | CGA TAA CAG TCT TGC CCC ACT T |
| *COL10* | ACG CTG AAC GAT ACC AAA TG | TGC TAT ACC TTT ACT CTT TAT GGT GTA |
| *GAPDH* | ACA TCG CTC AGA CAC CAT G | TGT AGT TGA GGT CAA TGA AGG G |
